# Sleep in Infancy Predicts Gender Specific Social-Emotional Problems in Toddlers

**DOI:** 10.3389/fped.2015.00042

**Published:** 2015-05-11

**Authors:** Janet Saenz, Ashley Yaugher, Gerianne M. Alexander

**Affiliations:** ^1^Department of Psychology, Texas A&M University, College Station, TX, USA; ^2^Division of Adolescent Medicine, Children’s Hospital Los Angeles, Los Angeles, CA, USA

**Keywords:** actigraphy, sleep, infants, toddlers, social-emotional, longitudinal studies

## Abstract

Despite strong evidence linking sleep to developmental outcomes, the longitudinal relationship between sleep and emotional well-being remains largely unknown. To address this gap in our knowledge, the current study examined sleep in infancy, measured via actigraphy, as a predictor of social-emotional problems in toddlers. A total of 47 children (29 males) were included in this longitudinal study. At time one, actigraphy measures of sleep were obtained from 3- to 4-month-old infants. At time two, parents rated their 18- to 24-month-old toddler’s social-emotional well-being using the Brief Infant Toddler Social Emotional Assessment. Results indicated that boys tended to have higher levels of externalizing behaviors than did girls. Additionally, boys with longer sleep durations also showed lower sleep efficiency. In girls, sleep duration in infancy was a significant predictor of autism spectrum disorder behaviors and approached significance as a predictor of externalizing problems in toddlerhood. Our findings are the first to show a relationship between sleep measured in infancy and autism spectrum disorder symptomatology measured in early childhood. They suggest that the etiology of social-emotional problems may differ between genders and raise the possibility that sleep/wake cycles may be differentially related to autism spectrum disorder symptoms in girls and boys.

## Introduction

Newborns spend the majority of their time asleep and undergo significant changes in their sleep/wake cycles during the first few years of life. During the first year of life, daytime sleep decreases, while total wake time increases (1). Additionally, total night time sleep and the length of the longest night time sleep period increase during the first 12 months of life (1). Although sleep continues to consolidate during the second year of life, sleep schedules (e.g., sleep duration) do not change significantly during this time (2). On average, sleep problems tend to decline from age four through mid-adolescence. However, individual differences persist and remain stable, such that those who have more sleep problems at age four also have more sleep problems during mid-adolescence (3).

Sleep in infancy has been associated with cognitive (4), physical (5), psychomotor (6), and temperament development (7) in early childhood. Sleep has also been shown to impact behavior and mood in typically developing children and adolescents (8). For example, diurnal sleep duration has been inversely correlated with emotional regulation in infancy (7), and self-reported sleepiness during adolescence has been associated with more anxiety, depression, and perceived negative health (9). Sleep duration has also been shown to moderate the association between maternal sensitivity and internalizing/externalizing symptoms in early childhood, such that maternal sensitivity is associated with less internalizing/externalizing symptoms in children who sleep more and unrelated in children who sleep less (10). Moreover, short sleep durations have been associated with more attention and externalizing problems, while irregularity in sleep duration has been associated with more internalizing problems in 8-year-old children (11). Additionally, sleep problems have been positively correlated with both increased conduct problems (12) and increased symptoms of anxiety/depression (3) in children and adolescents.

Generally, children diagnosed with psychological disorders experience more sleep problems than their typically developing peers (13). For example, preschool children diagnosed with autism spectrum disorder (ASD) (14), a disorder characterized by symptoms of social-communication deficits and stereotyped patterns of behavior ranging from low to high severity level (15, 16), have been shown to sleep less and have more variability in their sleep-wake cycles than typically developing children (17). Similarly, school-aged children diagnosed with attention deficit hyperactivity disorder (ADHD) experience more sleep impairments and variability in their sleep-wake cycles than do typically developing children (18, 19). While children diagnosed with a psychological disorder experience greater sleep difficulties than their typically developing peers, children diagnosed with comorbid conditions (e.g., ADHD/anxiety or ADHD/depression) experience even greater sleep difficulties than children diagnosed with a single condition (e.g., ADHD) (20). Likewise, the severity of sleep disturbances has been associated with the severity of psychopathology in adolescents, such that adolescents with multiple psychiatric disorders have more sleep problems compared to adolescents with only one psychiatric disorder (21).

Although many studies have investigated the association between sleep and psychological functioning in healthy and clinical samples, few studies have investigated the longitudinal relationship between these two constructs in early life. From these limited number of longitudinal studies, we know that parental reports of disrupted sleep patterns predict less optimal adjustment in preschool (22). Previous research also indicates that approximately one in four children with severe sleep problems in infancy, as measured by parental reports, will meet criteria for a diagnosis of ADHD at age 5 (23). This is consistent with other research which considers snoring, a specific type of sleep problem, in early infancy to be a risk factor for hyperactive behavior during toddlerhood (24). Furthermore, parent reported sleep problems at age four have also been shown to significantly predict more depression/anxiety, inattention/overactivity, and aggression in mid-adolescence (3).

Parents are typically not able to monitor infant sleep continuously throughout the night. Therefore, it is not surprising that parental reports of sleep patterns are often unreliable and non-specific (25–27). In contrast, actigraphy has been shown to be a valid and reliable measure of sleep when compared to polysomnography, the gold standard for sleep measurement (28, 29). The goal of the current study was to examine the relationship between sleep in infancy and social-emotional problems in toddlers using actigraphy. We hypothesized that decreased sleep duration and decreased sleep efficiency in infancy would be predictive of increased social-emotional problems during toddlerhood.

## Materials and Methods

### Participants

Participants included 47 children (29 males and 18 females) who took part in a longitudinal study investigating sex-linked behavior. For more information on the sample, please refer to the following articles (30–35). Signed informed consent was obtained from all parents prior to participation in the study. The Institutional Review Board (IRB) Human Subjects Committee approved procedures described in this paper.

### Measures

#### Sleep Measures

As sleep duration and sleep efficiency undergo rapid changes in the first year of life, to limit any confounding effects of age, we restricted our measurement of sleep to 3 to 4 months (*M* = 3.88 months, SD = 0.64 months). Infants’ sleep patterns were recorded for five consecutive days using actigraphs (Mini Mitter Actiwatch, AW64; Phillips Respironics, Bend, Oregon), small accelerometers that can be attached to the body and provide direct recordings of movements. In this technology, activity counts based on movement are generated for a specified epoch length, and each epoch is assessed as sleep or wake based on whether or not it exceeds an activity threshold. The manufacturer’s algorithm was set at a threshold of medium sensitivity (activity score of 40), and 30-s epoch lengths were used to calculate data across the 5 days. Study outcome variables included sleep duration (defined in minutes of the sleep period that were spent sleeping) and sleep efficiency (defined as the percentage of the sleep period that was spent sleeping), key variables that have been investigated in previous research, and have been shown to correlate strongly and moderately, respectively, with polysomnography (36–38). The small watch-like actigraphs were placed on infants’ legs and removed at the end of the 5-day assessment period.

#### Social-Emotional Problems

At 18 to 24 months (*M* = 19.82 months, SD = 2.07 months), parents completed the Brief Infant-Toddler Social and Emotional Assessment, which requires a fourth- to sixth-grade reading level and takes approximately 7 min to complete. The Brief Infant-Toddler Social and Emotional Assessment has high internal consistency, inter-rater reliability, test-retest reliability, and predictive validity (39). The 42-item questionnaire yields four subscales measuring externalizing problems (i.e., scores ranging from 0 to 12), internalizing problems (i.e., scores ranging from 0 to 16), dysregulation problems (i.e., scores ranging from 0 to 16), and autism spectrum behaviors (i.e., scores ranging from 0 to 17) (40). Data from these four subscales were previously examined in relation to hormone factors (35).

## Results

### Sex differences

A one-way ANOVA was conducted to determine if there was a significant difference between boys and girls in our variables of interest. No significant sex differences were noted in sleep duration (boys: *M* = 473.14 min, SD = 132.39 min vs. girls: *M* = 516.22 min, SD = 113.98 min, *d* = 0.35) or sleep efficiency (boys: *M* = 93.14, SD = 2.90 vs. girls: *M* = 92.11, SD = 2.82, *d* = 0.36), *F*_(1,45)_ = 1.30, 1.42, respectively. With regard to social-emotional problems, there was a trend for boys to have more externalizing problems than girls (boys: *M* = 2.48, SD = 1.43 vs. girls: *M* = 1.67, SD = 1.37, *d* = 0.58), *F*_(1,45)_ = 3.73, *p* = 0.06. However, internalizing problems (boys: *M* = 1.69, SD = 1.31 vs. girls: *M* = 1.56, SD = 1.34, *d* = 0.10), dysregulation problems (boys: *M* = 2.48, SD = 1.68 vs. girls: *M* = 3.17, SD = 2.33, *d* = 0.34), and autism spectrum behaviors (boys: *M* = 3.41, SD = 1.52 vs. girls: *M* = 3.89, SD = 2.80, *d* = 0.21) were not significantly different between boys and girls, *F*_(1,45)_ = 0.74, 0.25, 0.46, respectively (41).

### Correlations

Pearson Product Moment correlations were conducted to explore the association among sleep measures between boys and girls separately. In boys, the correlation between sleep duration and sleep efficiency approached significance, *r* (27) = −0.35, *p* = 0.06, such that boys who had longer sleep durations also had lower sleep efficiency. In girls, the correlation between sleep measures was not significant, *r* (16) = −0.01, *p* > 0.05.

### Regressions

To determine if sleep duration and sleep efficiency at 3 months significantly predicted social-emotional problems in toddlers, a simultaneous regression was conducted for externalizing problems, internalizing problems, dysregulation problems, and autism spectrum behaviors with both sleep efficiency and sleep duration as predictors. As recommended by previous researchers (42), regressions were conducted for boys and girls separately.

The regressions used to predict externalizing problems [*F* (2, 26) = 0.22, *p* > 0.05, *R*^2^ = 0.02], internalizing problems [*F* (2, 26) = 1.22, *p* > 0.05, *R*^2^ = 0.09], dysregulation problems [*F* (2, 26) = 0.19, *p* > 0.05, *R*^2^ = 0.02], and autism spectrum behaviors [*F* (2, 26) = 0.40, *p* > 0.05, *R*^2^ = 0.03] in boys were not significant and no main effects emerged (Table 1).

**Table 1:** **Summary of simultaneous multiple regressions with sleep duration and sleep efficiency predicting externalizing problems, internalizing problems, dysregulation problems, and autism spectrum behaviors in boys**.

	Externalizing problems (*n* = 29)^a^			Internalizing problems (*n* = 29)^b^			Dysregulation problems (*n * = 29)^c^			Autism spectrum behaviors (*n* = 29)^d^		
	*B*	SE	β	*B*	SE	β	*B*	SE	β	*B*	SE	β
Sleep duration	0.00	0.00	0.05	0.00	0.00	0.28	−0.00	0.00	−0.11	0.00	0.00	0.09
Sleep efficiency	−0.05	0.10	−0.10	0.11	0.09	0.23	−0.06	0.12	−0.10	−0.06	0.11	−0.12

*^a^*R*^2^ = 0.02, *F* (2, 26) = 0.22, *p* > 0.05*.

*^b^*R*^2^ = 0.09, *F* (2, 26) = 1.22, *p* > 0.05*.

*^c^*R*^2^ = 0.02, *F* (2, 26) = 0.19, *p* > 0.05*.

*^d^*R*^2^ = 0.03, *F* (2, 26) = 0.40, *p* > 0.05*.

*p < 0.05.

The overall regression used to predict externalizing problems in girls was not significant, *F* (2, 15) = 2.73, *p* = 0.098, *R*^2^ = 0.27. However, of the predictors investigated, sleep duration was approaching significance as a predictor of externalizing problems, β = −0.46, *t* (15) = −2.06, *p* = 0.058 (Table 2). That is, after controlling for sleep efficiency, a 1-min decrease in sleep duration resulted in a 0.005 point predicted increase in a child’s externalizing problems score. The regressions used to predict internalizing problems [*F* (2, 15) = 0.69, *p* > 0.05, *R*^2^ = 0.09] and dysregulation problems [*F* (2, 15) = 0.95, *p* > 0.05, *R*^2^ = 0.11] were also not significant, and no main effects emerged. Lastly, the regression used to predict autism spectrum behaviors in girls was not significant, *F* (2, 15) = 2.6, *p* > 0.05, *R*^2^ = 0.51. However, of the predictors investigated, sleep duration was a significant predictor of autism spectrum behaviors, β = −0.48, *t* (15) = −2.14, *p* < 0.05. That is, after controlling for sleep efficiency, a 1-min decrease in sleep duration resulted in a 0.01 point predicted increase in a child’s autism spectrum behaviors score.

**Table 2:** **Summary of simultaneous multiple regressions with sleep duration and sleep efficiency predicting externalizing problems, internalizing problems, dysregulation problems, and autism spectrum behaviors in girls**.

	Externalizing problems (*n* = 18)^a^			Internalizing problems (*n* = 18)^b^			Dysregulation problems (*n* = 18)^c^			Autism spectrum behaviors (*n* = 18)^d^		
	*B*	SE	β	*B*	SE	β	*B*	SE	β	*B*	SE	β
Sleep duration	−0.01	0.00	−0.46	−0.00	0.00	−0.14	0.00	0.01	0.07	−0.01	0.01	−0.48*
Sleep efficiency	−0.12	0.11	−0.25	−0.12	0.12	−0.26	0.27	0.20	0.33	0.17	0.22	0.17

*^a^*R*^2^ = 0.27, *F* (2, 15) = 2.73, *p* > 0.05*.

*^b^*R*^2^ = 0.09, *F* (2, 15) = 0.69, *p* > 0.05*.

*^c^*R*^2^ = 0.11, *F* (2, 15) = 0.95, *p* > 0.05*.

*^d^*R*^2^ = 0.51, *F* (2, 15) = 2.6, *p* > 0.05*.

*p < 0.05.

## Discussion

In this research, less sleep duration in infant girls across a period of 5 days was predictive of higher ASD scores on the BITSEA in toddlerhood. To our knowledge, these findings are the first to show a longitudinal relationship between sleep in infancy and ASD symptomatology in early childhood. Our data are also consistent with previous research showing that fewer hours of sleep per night, measured via parental report, exacerbate ASD symptoms in a sample of mostly male school-aged children (43). However, a positive relationship between sleep deficits and subclinical levels of behaviors associated with ASD was not found in boys in this investigation. This suggests that sleep/wake cycles in infancy may be differentially related to later social-emotional problems in girls and boys, a possibility that may inform understandings of sex differences in the development of ASD.

Autism spectrum disorder has a large comorbidity with other conditions, including sleeping problems (44, 45). In addition, children with ASD have higher rates of sleep problems than typically developing children (46, 47), with prevalence rates of sleeping problems in children with ASD typically ranging from 41 to 86% (48, 49). Our research showing less sleep in infancy is predictive of higher scores on measure of ASD symptoms suggests it may be useful to monitor the sleep of infants who are at risk for ASD and intervene when indicated. Others have speculated that sleep deprivation in early adulthood may cause long-term maladaptive changes in brain systems related to emotional reactions (e.g., frontal lobe, amygdala) (50). If so, the rapid brain development in infancy (51) may increase vulnerability to sleep deficits. Notably, behavioral interventions have been shown to improve sleep in typically developing children (52), as well as children with ASD (47). Melatonin has also been shown to improve sleep in typically developing children (53) and children with ASD (54, 55). Therefore, behavioral and pharmacological interventions may emerge as potential interventions in those at high risk for sleep problems in infancy and thereby reduce risk for social-emotional problems in childhood.

We also found a tendency for shorter sleep durations to predict higher levels of externalizing symptoms in girls at 18 months of age. This finding is noteworthy given that actigraphy measures of sleep duration in 5-year-olds have been negatively correlated with externalizing problems (e.g., aggression) (36), whereas parental reports of sleep have not (56). Other research showing parental reports of sleep patterns in typically developing children and children with psychological disorders (e.g., ASD, ADHD) are not frequently associated with actigraphy measured sleep patterns (27, 57–59), suggesting it may be informative to include actigraphy in future research on sleep and childhood psychopathology.

The use of a non-clinical sample limits the generalizability of results to children with clinically significant social-emotional problems. However, given an increasing recognition that many psychological disorders are extreme presentations of typical behavior (15), our findings may add to our understanding of the development of these symptoms at the low severity end of the diagnostic spectrums. Our study also utilized a brief screening questionnaire, intended to identify individuals who may be at-risk for developing problems in specific domains in the future. Although this measure has excellent predictive validity (60), it provides measures of symptoms characteristic of broad social-emotional problems (e.g., externalizing problems, internalizing problems, dysregulation problems, and autism spectrum behaviors). For that reason, it will be important to replicate our results in future research using high risk cohorts and information from multiple informants (e.g., self-report, parent-report, teacher-report, etc.) and multiple formats (e.g., clinical interviews, questionnaires, observations, etc.).

## Author Contributions

JS drafted the manuscript. All coauthors contributed to data analyses, interpretation of results, and revision of manuscript for intellectual content. The final version of the manuscript was approved by all coauthors. JS agreed to be accountable for all aspects of the work in ensuring that questions related to the accuracy or integrity of any part of the work are appropriately investigated and resolved.

## Conflict of Interest Statement

The authors declare that the research was conducted in the absence of any commercial or financial relationships that could be construed as a potential conflict of interest.
